# Atrial fibrillation as a risk factor for cognitive decline and dementia

**DOI:** 10.1093/eurheartj/ehx208

**Published:** 2017-04-29

**Authors:** Archana Singh-Manoux, Aurore Fayosse, Séverine Sabia, Marianne Canonico, Martin Bobak, Alexis Elbaz, Mika Kivimäki, Aline Dugravot

**Affiliations:** 1 INSERM, U1018, Centre for Research in Epidemiology and Population Health, Université Paris-Saclay., Hôpital Paul Brousse, Bât 15/16, 16 Avenue Paul Vaillant Couturier, VILLEJUIF CEDEX, 94807, France; 2 Department of Epidemiology and Public Health, University College London, 1-19 Torrington Place, London WC1E 6BT, UK

**Keywords:** Atrial fibrillation, Cognitive decline, Dementia, Ageing

## Abstract

**Aims:**

To assess whether AF is a risk factor for cognitive dysfunction we used prospective data on AF, repeat cognitive scores, and dementia incidence in adults followed over 45 to 85 years.

**Methods and results:**

Data are drawn from the Whitehall II study, *N* = 10 308 at study recruitment in 1985. A battery of cognitive tests was administered four times (1997–2013) to 7428 participants (414 cases of AF), aged 45–69 years in 1997. Compared with AF-free participants, those with longer exposure to AF (5, 10, or 15 years) experienced faster cognitive decline after adjustment for sociodemographic, behavioural, and chronic diseases (*P* for trend = 0.01). Incident stroke or coronary heart disease individually did not explain the excess cognitive decline; however, this relationship was impacted when considering them together (*P* for trend 0.09). Analysis of incident dementia (*N* = 274/9302 without AF; *N* = 50/912 with AF) showed AF was associated with higher risk of dementia in Cox regression adjusted for sociodemographic factors, health behaviours and chronic diseases [hazard ratio (HR): 1.87; 95% confidence interval (CI): 1.37, 2.55]. Multistate models showed AF to increase risk of dementia in those free of stroke (HR: 1.67; 95% CI: 1.17, 2.38) but not those free of stroke and coronary heart disease (HR: 1.29; 95% CI: 0.74, 2.24) over the follow-up.

**Conclusion:**

In adults aged 45–85 years AF is associated with accelerated cognitive decline and higher risk of dementia even at ages when AF incidence is low. At least in part, this was explained by incident cardiovascular disease in patients with AF.

## Introduction

The incidence of atrial fibrillation (AF), a type of cardiac arrhythmia which produces rapid and irregular heartbeat, rises steeply with age,^1^ and has major health consequences due to increased likelihood of thrombo-embolic complications and heart failure. The risk of stroke is increased as much as five-fold in patients with AF.^2^ Stroke is a risk factor for dementia,^3^^,^^4^ and there is consistent evidence that AF increases the risk of dementia, particularly in stroke patients.^5^ A recent meta-analysis reported associations of AF with cognitive impairment and dementia in those without stroke, although the associations were weaker than in stroke patients.^6^

Much of the evidence on AF and cognitive impairment, including studies in the recent systematic reviews,^5^^,^^6^ comes from data on the elderly among whom both conditions are more common, and their association might simply reflect manifestations of underlying systemic vascular disease. Disentangling the direction of association in studies on the elderly is complicated as bidirectional effects are common. For example, in older adults dementia is a risk factor for stroke and vice-versa.^7^^,^^8^ As the pathophysiological processes underlying dementia unfold over a very long period prior to clinical onset,^9^ further evidence of the importance of AF for cognitive decline and dementia needs to examine whether AF at younger ages increases risk of accelerated cognitive decline and dementia.

To address some of these complexities, we sought to examine associations of incident AF with subsequent cognitive decline over the 45 to 85 year age span. Our aim was to determine the potential dose-response association of duration of exposure to AF on cognitive decline, and whether stroke and coronary heart disease (CHD) subsequent to AF mediated this association. We also examined whether AF increased dementia risk, and the extent to which it was mediated by stroke and CHD.

## Methods

### Study population

The Whitehall II study is an ongoing cohort study of persons originally employed by the British civil service. A total of 10 308 persons (33% women), aged 35–55 years, were recruited to the study over the years 1985–1988.^10^ All participants responded to a comprehensive questionnaire and underwent a uniform, structured clinical evaluation, consisting of measures of anthropometry, cardiovascular and metabolic risk factors and disease. Since the baseline medical examination, follow-up examinations have taken place approximately every 5 years. Participant consent and research ethics approvals (University College London (UCL) ethics committee) are renewed at each contact; the latest approval was by the Joint UCL/UCLH Committee on the Ethics of Human Research (Committee Alpha), reference number 85/0938.

### Assessment of AF

Two sources were used: a twelve-lead resting ECG (Mingorec, Siemens Healthcare, Erlangen, Germany) at all 6 waves of data collection between 1985 and 2013, and interpreted for the presence of AF/flutter at the University of Glasgow (Prof. Macfarlane). Coding was carried out independently in duplicate by trained technicians; Minnesota codes 83x were used to identify cases of AF.^11^ The second source was the national hospital episode statistics (HES) database on hospital day cases and inpatients, using ICD9: 427.3 and ICD10: I48. The National Health Service (NHS) in the United Kingdom provides most of the health care, and record linkage is based on encrypted values of the NHS number, unique for each person.

### Cognitive function

The cognitive test battery was introduced to the study in 1997 and data are available at four assessments until 2013. The tests, described below, provide a comprehensive assessment of cognitive function and are appropriate for this population composed of individuals younger than in most studies on cognitive ageing.^12^

- *Memory* was assessed using a 20-word free recall test. Participants were presented a list of one or two syllable words at two second intervals and were then asked to recall in writing as many of the words in any order with two minutes to do so.
- *Reasoning*, assessed via the Alice Heim 4-I test which is composed of a series of 65 verbal and mathematical reasoning items of increasing difficulty.^13^ It tests inductive reasoning, measuring the ability to identify patterns and infer principles and rules. Participants had 10 minutes to do this section.
- *Verbal fluency*, measures of phonemic and semantic fluency were used.^14^ Participants were asked to recall in writing as many words beginning with ‘s’ (phonemic fluency) and as many animal names (semantic fluency) as they could. One minute was allowed for each test and the scores combined for the analysis.

A *global cognitive score* was created using all tests by first standardizing the raw scores to z-scores [mean = 0; standard deviation (SD) = 1] and then averaging and standardizing them.

### Dementia

We used comprehensive tracing of electronic health records using three databases: HES, Mental Health Services Data Set (MHSDS) and the mortality register. Record linkage until 3Ist of March 2015, using ICD-10 codes F00, F01, F02, F03, F05.1, G30, G31.0, G31.1, and G31.8 identified cases of dementia. MHSDS is a national database which contains information for persons in contact with mental health services in hospitals, outpatient clinics, and the community. Mortality data were drawn from the British national mortality register. The validity of dementia ascertainment in our study is supported by findings on changes in the global cognitive score, showing accelerated decline in global cognitive score in the 8–10 years before dementia diagnosis (Supplementary material online, *Figure S1*). This is in agreement with previous studies that used a ‘gold-standard’ dementia ascertainment procedure.^15^

### Covariates

Socio-demographic measures included age, sex, ethnicity (white, non-white), and education (lower secondary school or less, higher secondary school (usually achieved at age 18), and university or higher degree). Analyses were also adjusted for health behaviours assessed by questionnaire, including smoking (current-, ex-, never-smoker); alcohol consumption (units of alcohol consumed in a week: no/occasional alcohol consumption, moderate alcohol consumption (1–14 (21)units/week in women (men), and heavy alcohol consumption (≥14 (21) units in women (men)); physical activity categorized as active (≥2.5 h/week of moderate physical activity or ≥1 h/week of vigorous physical activity), inactive (<1 h/week of moderate and vigorous activity), and intermediate level of activity for all others; and dietary behaviour self-reported frequency of fruit and vegetable consumption (<once a day, once a day, >once a day).

Chronic diseases included were hypertension (systolic/diastolic 140/90 mmHg or antihypertensive medication), prevalent diabetes mellitus (fasting glucose ≥ 7.0 mmol/L, a 2-h post-load glucose ≥ 11.1 mmol/L, doctor-diagnosed diabetes, diabetes medication), heart failure (ICD codes: I50), coronary heart disease (CHD, ICD codes: I20–I25), stroke (ICD codes: I60–I64), and self-reported use of medication for cardiovascular disease.

### Statistical analysis

We examined associations between AF and participant characteristics in 1997–1999. Flow chart of persons included in the analyses described below is shown in Supplementary material online, *Figure S2*.

#### Analysis of cognitive decline

Mixed-effects models,^16^ with AF duration as a time varying covariate was used to estimate differences in cognitive decline between those without AF to those with AF exposure for 5, 10, and 15 years. The basic model (Model 1) contained time (here age, centred at 65 years), time squared, years since incident AF (0 = no AF, in years for those with AF), sex, ethnicity, education, and year of birth. Subsequent analyses (Model 2) were adjusted for health behaviours and chronic diseases in 1997–1999. Further analyses examined the mediating roles of stroke (Model 3), CHD (Model 4), and both stroke and CHD (Model 5) by including them as time varying covariates (1997 to 2013) in the analysis. The model yielded mean effects for AF duration; subsequently an interaction term with age and age^2^ was used to assess whether these effects varied as a function of age.

#### Analysis of incident dementia

We first used Cox regression to analyse the association of AF with incidence of dementia. Participants were followed from 1985 until the record of dementia, death, or March 31st 2015, whichever came first. Age was the time scale and analyses adjusted for sex, ethnicity, and education (Model 1) and then for health behaviours and chronic diseases at baseline (Model 2).

In subsequent analyses, we examined the mediating role of stroke and CHD over the follow-up in the association between AF and incidence of dementia. These analyses were carried out using multistate models with a Weibull distribution. These models are an extension of competing risks survival analysis, allowing simultaneous estimation of the risk associated with AF in a) the incidence of stroke, b) the risk of dementia in those with stroke, and c) the risk of dementia in those free of stroke. Age was used as the timescale, and models were adjusted for sociodemographic factors, health behaviours, and chronic diseases at baseline. We repeated the analyses replacing stroke with CHD and then with CVD (stroke and CHD). These analyses were undertaken using R (SmoothHazard); all other analyses used Stata version 14. A two-sided *P*-value < 0.05 was considered statistically significant.

## Results

### Analysis of cognitive decline

A total of 7428 participants were included in the analysis, characteristics in 1997–1999 presented in *Table 1*. A total of 43% of participants had data at all four assessments, 29% at three, 15% at two and 13% at only one assessment; a total of 414 cases of AF were used in the analysis.

**Table 1:** Sample characteristics at the start of cognitive testing (1997–1999)

	No AF	AF	P*
N	7014	414	
Male, %	69.7	84.3	<0.001
Age (years), M (SD)	55.5 (6.0)	58.8 (5.9)	<0.001
Education (<secondary school), %	44	45.7	0.64
Ethnicity (white), %	91.3	95.4	0.003
Current smoker, %	10.5	8.7	0.29
Heavy alcohol consumption,^a^ %	25	32.1	0.001
Poor diet,^b^ %	27.9	24.9	0.42
Physically inactive,^c^ %	18.2	13.5	0.004
Diabetes, %	4.3	5.6	0.21
Hypertension, %	28	43.7	<0.001
CVD, %	5.1	12.8	<0.001
CVD medication,^d^ %	6.1	14.7	<0.001
Heart failure, %	0.04	0	0.68

M, mean; SD, standard deviation; AF, atrial fibrillation; CVD, cardiovascular disease.

* *P* for heterogeneity.

a Heavy alcohol consumption was defined as 14+ units/week in women and 21+ units/week in men.

b Corresponds to fruit and vegetable consumption <once a day.

c Corresponds to <1 h/week of moderate and <1 h/week of vigorous physical activity.

d CVD medication includes antihypertensives, lipid lowering drugs, nitrates, antiplatelets, and anticoagulants.

**Figure 1:**
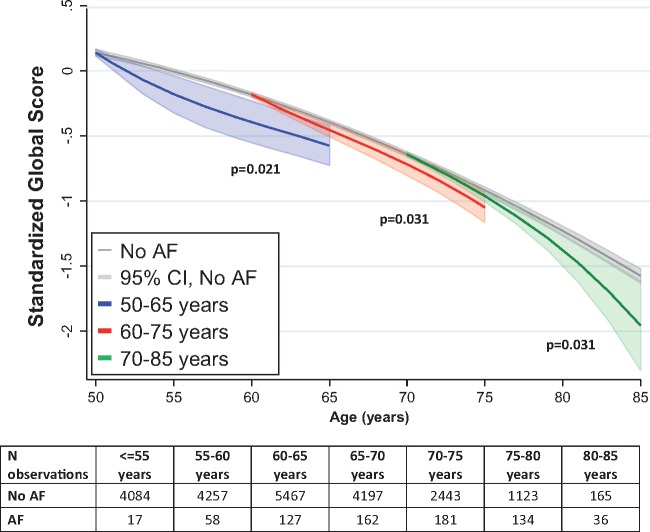
Decline in the global cognitive score as a function of atrial fibrillation (AF). *Analysis uses age as the time scale, adjusted for sex, education, and ethnicity. *P*-values represent the test for trend for greater effects on cognitive decline in longer exposure to AF.

The age range of participants at the beginning and end of follow-up was 45–69 years and 61–83 years; mean follow-up was 14.7 years and annual decline in global cognitive function was −0.050 SD (−0.054, −0.046). Excess cognitive decline, averaged across all age-groups, in analysis adjusted for all confounders (Model 2) was greater in those with AF for longer, *P* = 0.01 (*Table 2* and *Figure 1*). Incident stroke (Model 3), or CHD (Model 4) over the follow-up did not explain the excess decline. However, when both stroke and CHD (Model 5) were taken into account, the estimate for excess cognitive decline in those with AF was no longer statistically significant. The interaction term to test whether the effect of AF duration on cognitive decline changed with age did not suggest differences (*P* > 0.05), despite stronger associations in the youngest and oldest participants (*Table 2*). Furthermore, consideration of the incidence of stroke and CHD (model 5) attenuated associations in all but the youngest group (15 years difference in decline = −0.27; 95% CI: −0.51, −0.03).

**Table 2:** Estimates of decline in the global cognitive score over 15 years in those with atrial fibrillation (AF) compared with those without AF

		Covariates at baseline		Stroke/CHD over the follow-up		
		Model 1	Model 2 Model 1+all covariates	Model 3 Model 2 + Stroke	Model 4 Model 2 + CHD	Model 5 Model 2 + CVD
		Beta (95% CI)	Beta (95% CI)	Beta (95% CI)	Beta (95% CI)	beta (95% CI)
Mean (across all age-groups) 15 year Cognitive Decline						
NO AF		Ref.	Ref.	Ref.	Ref.	Ref.
additional decline when AF for 5 years		−0.05 (−0.09, −0.02)*	−0.05 (−0.09, −0.01)*	−0.04 (−0.08, −0.01)*	−0.04 (−0.08, −0.002)*	−0.03 (−0.07, 0.005)
additional decline when AF for 10 years		−0.11 (−0.18, −0.03)*	−0.10 (−0.17, −0.02)*	−0.09 (−0.16, −0.01)*	−0.08 (−0.15, −0.004)*	−0.07 (−0.14, 0.01)
additional decline when AF for 15 years		−0.16 (−0.27, −0.05)*	−0.15 (−0.26, −0.04)*	−0.13 (−0.24, −0.02)*	−0.12 (−0.23, −0.01)*	−0.10 (−0.21, 0.01)
*P* for trend		0.005	0.01	0.02	0.04	*0.09*
15 year Cognitive Decline as a function of age						
CURRENT AGE						
60 years	Decline between 45 and 60 year, NO AF	−0.43 (−0.49, −0.37)*				
	additional decline when AF at 45 years	−0.31 (−0.56, −0.07)*	−0.30 (−0.54, −0.06)*	−0.27 (−0.51, −0.04)*	−0.29 (−0.53, −0.06)*	−0.27 (−0.51, −0.03)*
65 years	Decline between 50 and 65 year, NO AF	−0.53 (−0.57, −0.49)*				
	additional decline when AF at 50 years	−0.18 (−0.33, −0.03)*	−0.16 (−0.32, −0.01)*	−0.14 (−0.29, 0.01)	−0.17 (−0.32, −0.02)*	−0.14 (−0.30, 0.01)
70 years	Decline between 55 and 70 year, NO AF	−0.63 (−0.66, −0.59)*				
	additional decline when AF at 55 years	−0.12 (−0.24, 0.01)	−0.10 (−0.23, 0.02)	−0.08 (−0.21, 0.04)	−0.10 (−0.22, 0.03)	−0.07 (−0.20, 0.05)
75 years	Decline between 60 and 75 years, NO AF	−0.73 (−0.77, −0.68)*				
	additional decline when AF at 60 years	−0.13 (−0.25, −0.01)*	−0.12 (−0.24, 0.001)	−0.10 (−0.22, 0.01)	−0.08 (−0.20, 0.04)	−0.06 (−0.18, 0.06)
80 years	Decline between 65 and 80 years, NO AF	−0.83 (−0.90, −0.76)*				
	additional decline when AF at 65 years	−0.22 (−0.40, −0.04)*	−0.20 (−0.38, −0.02)*	−0.20 (−0.38, −0.02)*	−0.12 (−0.31, 0.06)	−0.11 (−0.30, 0.08)
85 years	Decline between 70 and 85 years, NO AF	−0.93 (−1.02, −0.83)*				
	additional decline when AF at 70 years	−0.38 (−0.73, −0.03)*	−0.36 (−0.71, −0.01)*	−0.38 (−0.73, −0.03)*	−0.22 (−0.58, 0.14)	−0.21 (−0.57, 0.15)
Interaction between AF duration and age, *P*		0.16	0.17	0.14	0.22	*0.23*

Participants aged 45–69 years in 1997–1999 were followed until 2012–2013, mean follow-up 14.7 years. Estimates are for decline over 15 years.

Total *N* = 7428, atrial fibrillation, *N* = 414. *P < 0.05; CHD, coronary heart disease; CVD, cardiovascular disease (Stroke or CHD).

Model 1: Analysis uses age as the time-scale, adjusted for sex, education, and ethnicity.

Model 2: Model 1 + alcohol consumption, smoking, physical activity, diet, diabetes, hypertension, heart failure, CVD (stroke or CHD) and CVD medication at baseline (1997–1999).

Model 3: Model 2 + time-dependent Stroke (1997–2013), *N* = 109.

Model 4: Model 2 + time-dependent CHD (1997–2013), *N* = 1120.

Model 5: Model 2 + time-dependent CVD (1997–2013), *N* = 1182.

Supplementary material online, *Tables S1* and *S2* and *Figure S3* show these results separately for the tests of memory, reasoning, and fluency; there were no associations with memory, even in the minimally adjusted model (Supplementary material online, *Table S1*).

### Analysis of dementia

Of a total of 10 214 persons, followed over a mean 26.6 years, there were 912 cases of incident AF and 324 cases of dementia; 73% of the latter recorded in the last 5 years of follow-up. The mean (SD) age at incident AF and dementia diagnosis was 68.5 (7.7) and 74.9 (5.4) years, respectively. *Table 3* shows that in analysis adjusted for all confounders (Model 2), those with AF had 87% excess risk (95% CI: 1.37, 2.55) of dementia. The association was somewhat stronger, albeit not statistically significant, in those with incident AF before 70 years (*Table 3*).

**Table 3:** Association of atrial fibrillation (AF) with incidence of dementia

			Model 1	Model 2
Atrial fibrillation	*N* total	*N* cases	HR (95% CI)	HR (95% CI)
No	9302	274	1.00	1.00
Yes	912	50	1.93 (1.42, 2.63)	1.87 (1.37, 2.55)
Analysis stratified by age of onset of AF				
No	9302	274	1.00	1.00
AF before age 70	500	21	2.15 (1.37, 3.37)	2.11 (1.35, 3.32)
AF after age 70	412	29	1.79 (1.20, 2.65)	1.72 (1.15, 2.55)
*P* for interaction			0.50	0.45

Model 1: Analysis adjusted for age, sex, education, and ethnicity.

Model 2: Model 1 + alcohol consumption, smoking, physical activity, diet, diabetes, hypertension, heart failure, CVD (stroke or CHD), and CVD medication at baseline.

**Figure 2:**
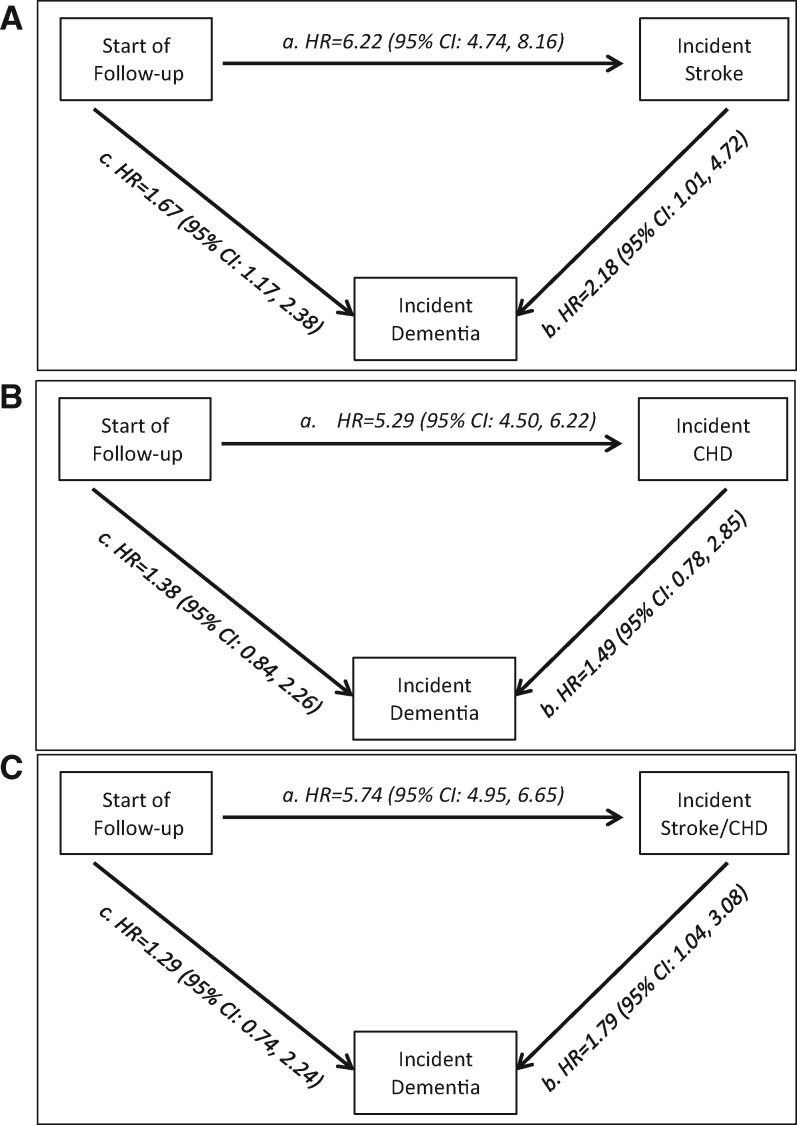
Multistate models for the role of atrial fibrillation in transitions* to stroke (*A*), CHD (*B*), stroke or CHD (*C*), and dementia. *Role of AF (time varying) in the risk of transitions from: (a) Healthy to stroke (*A*), CHD (*B*), and CVD (stroke or CHD, *C*); (b) Stroke (*A*, N (AF/Stroke) = 78/352), CHD (*B*, N (AF/CHD) = 179/1790), and CVD (Stroke or CHD, *C*, N (AF/CVD) = 217/2024) to dementia; (c) Healthy to dementia in those free of Stroke (*A*), CHD (*B*), and CVD (Stroke or CHD, *C*). Analyses with age as timescale and adjusted for sex, education, ethnicity, alcohol consumption, smoking, physical activity, diet, diabetes, hypertension, heart failure, CVD, and CVD medication at baseline.

In multistate models, AF was associated with a 6.22 times increased risk of stroke (95% CI: 4.74, 8.16) and its association with dementia was not fully explained by stroke as demonstrated by the increased risk of dementia in those free of stroke (HR = 1.67; 95% CI: 1.17, 2.38), *Figure 2A*. Further analysis showed AF to increase risk of CHD (HR = 5.29; 95% CI: 4.50, 6.22; *Figure 2B*) and CVD (HR = 5.74; 95% CI: 4.95, 6.65; *Figure 2C*). The analysis of CVD (*Figure 2C*) shows that the association between AF and dementia was present in those with CVD (HR = 1.79; 95% CI: 1.04, 3.08) but not in those free of CVD (HR = 1.29; 95% CI: 0.74, 2.24).

## Discussion

Our study of cognitive decline over 15 years in adults aged 45–69 years at the start of follow-up shows greater cognitive decline in those with longer exposure to AF. Stroke occurring after the onset on AF did not explain this excess decline but when both stroke and CHD over the follow-up were taken into account the association between duration of exposure to AF and cognitive decline was no longer statistically significant. This finding was replicated in analysis of dementia where stroke explained only part of the association of AF with dementia. Importantly, these findings relate to incident AF in relatively young adults as more than two thirds of AF cases in our analysis occurred before 75 years of age.

AF is a common disorder in the elderly,^17^ its prevalence increases with age, doubling every decade of life after the age of 50 years to reach 10–20% after the age of 80 years. As the prevalence of dementia also increases with age, its association with AF has sometimes been attributed to common age-related mechanisms. However, increasing evidence of associations with cognitive decline suggests that AF may indeed be a risk factor for cognitive dysfunction.^18^ To the best of our knowledge, few studies have been able to use serial cognitive testing, starting in mid-life to assess the impact of AF. We show that even in adults aged 60 years, those with incident AF at age 50 and 55 years had accelerated cognitive decline.

The effect of duration of AF has been examined in relation to dementia where the associations appear to be stronger in those with younger age of AF onset.^19–21^ Stronger effects associated with longer duration of AF have also been seen for pre-stroke cognitive impairment,^8^ and total brain and grey matter volume.^22^ AF is associated with cerebral hypoperfusion and is a known cause of embolic stroke, from thrombus originating in the atrial appendage. It is possible that longer duration of exposure allows a greater time window for damage from chronic hypoperfusion and development of emboli and cardiac failure. AF alters atrial size, substrate, and cardiac function, which develop very early after diagnosis, and increases the risk of macro- and micro-cerebral ischaemic events.

The association of AF with cognitive impairment,^6^^,^^23^cognitive decline,^24^ hippocampal atrophy,^25^and dementia,^26^ appears to be independent of stroke history. We extended these findings by considering stroke occurring after AF to show that it does not explain associations with cognitive decline and dementia. A third of those with AF have silent infarction^27^ and cerebrovascular thrombo-embolism together with global brain hypoperfusion due to impaired cardiac haemodynamics, may account for the increased risk of developing dementia in AF patients. Our findings highlight the importance of CVD in the association of AF with cognitive outcomes. The association of AF with dementia in our study is similar to that in previous studies,^20^^,^^26^ particularly studies that examined AF onset at younger ages.^19^ It remains unclear whether it affects risk of all types of dementia; one study found evidence of stronger associations with Alzheimer’s disease,^2^^0^ another showed the type of dementia not to matter,^19^ while MRI data associations with lower total grey matter but not neurodegenerative changes characteristic of Alzheimer’s disease.^27^

The main strengths of our study include the large sample size, consideration of both cognitive decline and dementia, modelling of time lived with AF, and explicit analysis of the role played by stroke and CHD after AF onset—an important consideration given the strong association of AF with these conditions. Our results on the interaction effects with age suggest that that the effect of AF on cognitive decline is not confined to older ages. We present results using the global cognitive score, allowing replication across studies in the future. We have previously shown the risk factor-CHD associations in our study to be similar to that in a general population study,^28^ suggesting that the present findings are generalizable.

Limitations of the study include the method of dementia ascertainment, which has high specificity but is unlikely to be sensitive. The fact that dementia tracing was available on all participants in the study allows the study results to be free from attrition and selection biases that are common in studies on older adults. Furthermore, the AF-CVD association in our study was similar to that in other studies,^2^ suggesting that case definitions for both conditions are valid. We were not able to distinguish paroxysmal from persistent AF, in previous studies the latter was more strongly associated with silent cerebral ischaemia and worse cognitive function,^23^ and higher incidence of adverse cardiovascular outcomes even in patients treated by anticoagulated therapy.^29^ We were also limited by the smaller number of patients with AF, particularly in subgroup analysis (e.g. those with incident CVD) that resulted in broad confidence limits.

In sum, AF is the most common arrhythmia and its most well established consequence is stroke. With ageing of populations its impact on cognitive impairment and dementia has become increasingly important and understanding this association has relevance for the development of preventive and therapeutic strategies.^30^ AF can be treated with antiarrhythmic medication, cardioversion, or catheter ablation. Furthermore, thrombo-prophylaxis with oral anticoagulation is effective in reducing stroke risk, whether the same is true for dementia is unknown. The present longitudinal study shows that early onset AF and its duration matters for cognitive decline and dementia and highlight the importance of effectively treating cardiovascular disease in AF patients.^1^ In those with early age of AF onset, a longer exposure period might lead to changes that produce greater neuronal injury and loss, possibly due to the interaction of degenerative and vascular changes.

## Supplementary material

Supplementary material is available at *European Heart Journal* online.

## Funding

The Whitehall II study is supported by grants from the US National Institute on Aging (R01AG013196; R01AG034454); the UK Medical Research Council (MRC K013351). M.K. is supported by the MRC and NordForsk. MRC (MR/K013351/1) via a prepayment account with UCL.

**Conflict of interest**: none declared.

## Supplementary Material

Supplementary Figures and Tables

## References

[1] Kirchhof P, Benussi S, Kotecha D, Ahlsson A, Atar D, Casadei B, Castella M, Diener HC, Heidbuchel H, Hendriks J, Hindricks G, Manolis AS, Oldgren J, Popescu BA, Schotten U, Van Putte B, Vardas P, Agewall S, Camm J, Baron Esquivias G, Budts W, Carerj S, Casselman F, Coca A, De Caterina R, Deftereos S, Dobrev D, Ferro JM, Filippatos G, Fitzsimons D, Gorenek B, Guenoun M, Hohnloser SH, Kolh P, Lip GY, Manolis A, McMurray J, Ponikowski P, Rosenhek R, Ruschitzka F, Savelieva I, Sharma S, Suwalski P, Tamargo JL, Taylor CJ, Van Gelder IC, Voors AA, Windecker S, Zamorano JL, Zeppenfeld K. 2016 ESC Guidelines for the management of atrial fibrillation developed in collaboration with EACTS. Eur Heart J 2016;37:2893–2962. 27567408 10.1093/eurheartj/ehw210

[2] Wolf PA, Abbott RD, Kannel WB. Atrial fibrillation as an independent risk factor for stroke: the Framingham Study. Stroke 1991;22:983–988. 1866765 10.1161/01.str.22.8.983

[3] Leys D, Henon H, Mackowiak-Cordoliani MA, Pasquier F. Poststroke dementia. Lancet Neurol 2005;4:752–759. 16239182 10.1016/S1474-4422(05)70221-0

[4] Pendlebury ST, Rothwell PM. Prevalence, incidence, and factors associated with pre-stroke and post-stroke dementia: a systematic review and meta-analysis. Lancet Neurol 2009;8:1006–1018. 19782001 10.1016/S1474-4422(09)70236-4

[5] Kwok CS, Loke YK, Hale R, Potter JF, Myint PK. Atrial fibrillation and incidence of dementia: a systematic review and meta-analysis. Neurology 2011;76:914–922. 21383328 10.1212/WNL.0b013e31820f2e38

[6] Kalantarian S, Stern TA, Mansour M, Ruskin JN. Cognitive impairment associated with atrial fibrillation: a meta-analysis. Ann Intern Med 2013;158:338–346. 23460057 10.7326/0003-4819-158-5-201303050-00007 PMC4465526

[7] Imfeld P, Bodmer M, Schuerch M, Jick SS, Meier CR. Risk of incident stroke in patients with Alzheimer disease or vascular dementia. Neurology 2013;81:910–919. 23902701 10.1212/WNL.0b013e3182a35151

[8] Horstmann S, Rizos T, Rauch G, Fuchs M, Arden C, Veltkamp R. Atrial fibrillation and prestroke cognitive impairment in stroke. J Neurol 2014;261:546–553. 24413641 10.1007/s00415-013-7233-3

[9] Jack CR Jr, Knopman DS, Jagust WJ, Petersen RC, Weiner MW, Aisen PS, Shaw LM, Vemuri P, Wiste HJ, Weigand SD, Lesnick TG, Pankratz VS, Donohue MC, Trojanowski JQ. Tracking pathophysiological processes in Alzheimer's disease: an updated hypothetical model of dynamic biomarkers. Lancet Neurol 2013;12:207–216. 23332364 10.1016/S1474-4422(12)70291-0 PMC3622225

[10] Marmot MG, Smith GD, Stansfeld S, Patel C, North F, Head J, White I, Brunner E, Feeney A. Health inequalities among British civil servants: the Whitehall II study. Lancet 1991;337:1387–1393. 1674771 10.1016/0140-6736(91)93068-k

[11] Prineas RJ, Crown RS, Blackburn H, The Minnesota Code Manual of Electrocardiographic Findings: Standards and Procedures for Measurement and Classification. Bristol, UK: John Wright; 1982.

[12] Singh-Manoux A, Kivimaki M, Glymour MM, Elbaz A, Berr C, Ebmeier KP, Ferrie JE, Dugravot A. Timing of onset of cognitive decline: results from Whitehall II prospective cohort study. Bmj 2012;344:d7622. 22223828 10.1136/bmj.d7622 PMC3281313

[13] Heim AW, AH 4 Group Test of General Intelligence. Windsor, UK: NFER-Nelson Publishing Company Ltd; 1970.

[14] Borkowski JG, Benton AL, Spreen O. Word fluency and brain damage. Neuropsychologica 1967;5:135–140.

[15] Amieva H, Le GM, Millet X, Orgogozo JM, Peres K, Barberger-Gateau P, Jacqmin-Gadda H, Dartigues JF. Prodromal Alzheimer's disease: successive emergence of the clinical symptoms. Ann Neurol 2008;64:492–498. 19067364 10.1002/ana.21509

[16] Fitzmaurice GM, Laird NM, Ware JH. Applied Longitudinal Analysis. Hoboken, New Jersey: Wiley; 2004.

[17] Lloyd-Jones DM, Wang TJ, Leip EP, Larson MG, Levy D, Vasan RS, D'agostino RB, Massaro JM, Beiser A, Wolf PA, Benjamin EJ. Lifetime risk for development of atrial fibrillation: the Framingham Heart Study. Circulation 2004;110:1042–1046. 15313941 10.1161/01.CIR.0000140263.20897.42

[18] Thacker EL, McKnight B, Psaty BM, Longstreth WT Jr, Sitlani CM, Dublin S, Arnold AM, Fitzpatrick AL, Gottesman RF, Heckbert SR. Atrial fibrillation and cognitive decline: a longitudinal cohort study. Neurology 2013;81:119–125. 23739229 10.1212/WNL.0b013e31829a33d1 PMC3770176

[19] de Bruijn RF, Heeringa J, Wolters FJ, Franco OH, Stricker BH, Hofman A, Koudstaal PJ, Ikram MA. Association between atrial fibrillation and dementia in the general population. JAMA Neurol 2015;72:1288–1294. 26389654 10.1001/jamaneurol.2015.2161

[20] Ott A, Breteler MM, de Bruyne MC, van Harskamp F, Grobbee DE, Hofman A. Atrial fibrillation and dementia in a population-based study. The Rotterdam Study. Stroke 1997;28:316–321. 9040682 10.1161/01.str.28.2.316

[21] Jacobs V, Cutler MJ, Day JD, Bunch TJ. Atrial fibrillation and dementia. Trends Cardiovasc Med 2015;25:44–51. 25442732 10.1016/j.tcm.2014.09.002

[22] Stefansdottir H, Arnar DO, Aspelund T, Sigurdsson S, Jonsdottir MK, Hjaltason H, Launer LJ, Gudnason V. Atrial fibrillation is associated with reduced brain volume and cognitive function independent of cerebral infarcts. Stroke 2013;44:1020–1025. 23444303 10.1161/STROKEAHA.12.679381 PMC3632359

[23] Gaita F, Corsinovi L, Anselmino M, Raimondo C, Pianelli M, Toso E, Bergamasco L, Boffano C, Valentini MC, Cesarani F, Scaglione M. Prevalence of silent cerebral ischemia in paroxysmal and persistent atrial fibrillation and correlation with cognitive function. J Am Coll Cardiol 2013;62:1990–1997. 23850917 10.1016/j.jacc.2013.05.074

[24] Chen LY, Lopez FL, Gottesman RF, Huxley RR, Agarwal SK, Loehr L, Mosley T, Alonso A. Atrial fibrillation and cognitive decline-the role of subclinical cerebral infarcts: the atherosclerosis risk in communities study. Stroke 2014;45:2568–2574. 25052319 10.1161/STROKEAHA.114.005243 PMC4146651

[25] Knecht S, Oelschlager C, Duning T, Lohmann H, Albers J, Stehling C, Heindel W, Breithardt G, Berger K, Ringelstein EB, Kirchhof P, Wersching H. Atrial fibrillation in stroke-free patients is associated with memory impairment and hippocampal atrophy. Eur Heart J 2008;29:2125–2132. 18667399 10.1093/eurheartj/ehn341

[26] Santangeli P, Di Biase L, Bai R, Mohanty S, Pump A, Cereceda Brantes M, Horton R, Burkhardt JD, Lakkireddy D, Reddy YM, Casella M, Dello Russo A, Tondo C, Natale A. Atrial fibrillation and the risk of incident dementia: a meta-analysis. Heart Rhythm 2012;9:1761–1768. 22863685 10.1016/j.hrthm.2012.07.026

[27] Graff-Radford J, Madhavan M, Vemuri P, Rabinstein AA, Cha RH, Mielke MM, Kantarci K, Lowe V, Senjem ML, Gunter JL, Knopman DS, Petersen RC, Jack CR Jr, Roberts RO. Atrial fibrillation, cognitive impairment, and neuroimaging. Alzheimers Dement 2016;12:391–398. 26607820 10.1016/j.jalz.2015.08.164 PMC4841716

[28] Batty GD, Shipley M, Tabak A, Singh-Manoux A, Brunner E, Britton A, Kivimaki M. Generalizability of occupational cohort study findings. Epidemiology 2014;25:932–933. 25265141 10.1097/EDE.0000000000000184

[29] Senoo K, Lip GY, Lane DA, Buller HR, Kotecha D. Residual risk of stroke and death in anticoagulated patients according to the type of atrial fibrillation: AMADEUS trial. Stroke 2015;46:2523–2528. 26205373 10.1161/STROKEAHA.115.009487

[30] Poggesi A, Inzitari D, Pantoni L. Atrial fibrillation and cognition: epidemiological data and possible mechanisms. Stroke 2015;46:3316–3321. 26396028 10.1161/STROKEAHA.115.008225

