# Supplementary material for: Atrial fibrillation as a risk factor for cognitive decline and dementia
Source: Eur Heart J. 2017 Apr 29;38(34):2612–8. doi: 10.1093/eurheartj/ehx208 (PMC5837240; doi:10.1093/eurheartj/ehx208)
Supplement: Supplementary Figures and Tables [file af_and_cognition_suppl_r2_ehx208.docx]

**SUPPLEMENTARY DATA**

**Supplementary Figure S1. Trajectory of global cognitive score in dementia cases and other participants in the years leading to dementia diagnosis.**

**Difference in trajectory, p<0.0001**

|  | **Number of observations in the analysis** | | | | | |
| --- | --- | --- | --- | --- | --- | --- |
| **Years** | | **-18 to -16** | **-16 to -12** | **-12 to-8** | **-8 to-4** | **-4 to 0** |
| **Dementia free (N=7311)** | | 5192 | 2489 | 3714 | 5938 | 4777 |
| **Dementia cases (N=195)** | | 31 | 81 | 119 | 110 | 89 |

**Supplementary Figure S2. Flow chart for analysis of cognitive decline and dementia.**

**1985-1988**

Age 35-55 years

N at baseline = 10,308

N incident AF =13
N incident dementia =0

**1991-1993**

Age 40-64 years

N incident AF =4
N incident dementia =0

N incident AF =35
N incident dementia =1

N incident AF =139
N incident dementia =14

N incident AF =269
N incident dementia =43

**Dementia**

N = 10,308 with dementia status

N = 94 missing data on covariates

**N in analysis = 10,214**

**(Incident AF = 912)**

**(Incident Dementia = 324)**

N incident AF =161
N incident dementia =134

**End of follow-up:**

**March 31^st^ 2015**

N incident AF =321
N incident dementia =137

**2012-2013**

Age 60-83 years

N cognitive data = 4,484

**2007-2009**

Age 55-79 years

N cognitive data = 5,960

**2002-2004**

Age 50-74 years

N cognitive data = 6,253

**Cognitive trajectories**

N = 7,506 with at least 1 wave of cognitive data

N = 78 missing data on covariates

**N in analysis = 7,428**

**(Incident AF = 414)**

**1997-1999**

Age 45-69 years

N cognitive data = 5,844

**Supplementary Figure S3. Decline in memory, reasoning and fluency as a function of atrial fibrillation (AF).**

*Analysis uses age as the time-scale, adjusted for sex, education, and ethnicity.

**Supplementary Table S1. Estimates of cognitive decline over 15 years^†^ in those with atrial fibrillation (AF) compared to those without AF.**

**Analysis adjusted for sex, ethnicity and education (Model 1).**

|  |  | **Memory** | **Reasoning** | **Fluency** |
| --- | --- | --- | --- | --- |
|  |  | **beta (95% CI)** | **beta (95% CI)** | **beta (95% CI)** |
| **Mean (across all age-groups) 15 year Cognitive Decline** | | |  |  |
|  | **NO AF** | **Ref.** | **Ref.** | **Ref.** |
|  | additional decline: 5y after incident AF | -0.04 (-0.09, 0.01) | -0.03 (-0.07, -0.001)* | -0.05 (-0.09, -0.01)* |
|  | additional decline: 10y after incident AF | -0.08 (-0.18, 0.02) | -0.07 (-0.14, -0.001)* | -0.10 (-0.18, -0.02)* |
|  | additional decline: 15y after incident AF | -0.12 (-0.27, 0.03) | -0.10 (-0.21, -0.002)* | -0.15 (-0.27, -0.03)* |
|  | ***P for trend*** | ***0.11*** | ***0.045*** | ***0.01*** |
| **15 year Cognitive Decline as a function of age**  **CURRENT AGE** | |  |  |  |
| **60 years** | **Decline between 45-60 year, NO AF** | **-0.18 (-0.28, -0.08)*** | ***-0.25 (-0.30, -0.19)**** | ***-0.49 (-0.56, -0.42)**** |
|  | additional decline when AF at 45y | -0.26 (-0.59, 0.06) | -0.23 (-0.45, -0.01)* | -0.25 (-0.51, 0.01) |
| **65 years** | **Decline between 50-65 year, NO AF** | **-0.31 (-0.37, -0.24)*** | ***-0.33 (-0.36, -0.29)**** | ***-0.54 (-0.58, -0.49)**** |
|  | additional decline: incident AF at 50y | -0.21 (-0.42, -0.01)* | -0.11 (-0.25, 0.03) | -0.13 (-0.29, 0.03) |
| **70 years** | **Decline between 55-70 year, NO AF** | **-0.44 (-0.49, -0.39)*** | ***-0.41 (-0.44, -0.38)**** | ***-0.59 (-0.62, -0.55)**** |
|  | additional decline: incident AF at 55y | -0.15 (-0.33, 0.03) | -0.06 (-0.18, 0.06) | -0.09 (-0.23, 0.05) |
| **75 years** | **Decline between 60-75 years, NO AF** | **-0.56 (-0.64, -0.49)*** | ***-0.50 (-0.54, -0.45)**** | ***-0.63 (-0.69, -0.58)**** |
|  | additional decline: incident AF at 60y | -0.08 (-0.25, 0.10) | -0.08 (-0.19, 0.02) | -0.14 (-0.26, -0.01)* |
| **80 years** | **Decline between 65-80 years, NO AF** | **-0.69 (-0.80, -0.58)*** | ***-0.58 (-0.64, -0.52)**** | ***-0.68 (-0.76, -0.61)**** |
|  | additional decline: incident AF at 65y | 0.004 (-0.27, 0.28) | -0.18 (-0.34, -0.02)* | -0.26 (-0.46, -0.07)* |
| **85 years** | **Decline between 70-85 years, NO AF** | **-0.82 (-0.97, -0.67)*** | ***-0.66 (-0.75, -0.58)**** | ***-0.73 (-0.84, -0.63)**** |
|  | additional decline: incident AF at 70y | 0.09 (-0.47, 0.65) | -0.34 (-0.65, -0.04)* | -0.48 (-0.87, -0.09)* |
| ***Interaction between AF duration and age, p*** | | ***0.45*** | ***0.12*** | ***0.17*** |

*p<0.05

^†^ Participants aged 45-69 years in 1997-1999 were followed until 2012-2013, mean follow-up 14.7 years. Estimates are for decline over 15 years.

Analysis uses age as the time-scale; adjusted for sex, education, and ethnicity.

**Supplementary Table S2. Estimates of decline in the global cognitive score over 15 years^†^ in those with atrial fibrillation (AF) compared to those without AF.**

**Analysis adjusted for covariates at baseline and CVD over the follow-up (Model 5).**

|  |  | **Memory** | **Reasoning** | **Fluency** |  |
| --- | --- | --- | --- | --- | --- |
|  |  | **beta (95% CI)** | **beta (95% CI)** | **beta (95% CI)** |  |
| **Mean (across all age-groups) 15 year Cognitive Decline** | | |  |  |  |
|  | **NO AF** | **Ref.** | **Ref.** | **Ref.** |  |
|  | additional decline: 5y after incident AF | -0.03 (-0.08, 0.02) | -0.02 (-0.06, 0.01) | -0.03 (-0.07, 0.01) |  |
|  | additional decline: 10y after incident AF | -0.06 (-0.16, 0.05) | -0.04 (-0.11, 0.03) | -0.06 (-0.14, 0.02) |  |
|  | additional decline: 15y after incident AF | -0.09 (-0.24, 0.07) | -0.06 (-0.17, 0.04) | -0.09 (-0.20, 0.03) |  |
|  | ***P for trend*** | ***0.28*** | ***0.24*** | ***0.16*** |  |
| **15 year Cognitive Decline as a function of age**  **CURRENT AGE** | |  |  |  |  |
| **60 years** | **Decline between 45-60 year, NO AF** | ***-0.15 (-0.37, 0.06)*** | ***-0.28 (-0.40, -0.16)**** | ***-0.39 (-0.55, -0.24)**** |  |
|  | additional decline when AF at 45y | -0.22 (-0.54, 0.10) | -0.20 (-0.41, 0.02) | -0.22 (-0.48, 0.04) |  |
| **65 years** | **Decline between 50-65 year, NO AF** | ***-0.31 (-0.45, -0.17)**** | ***-0.35 (-0.43, -0.27)**** | ***-0.48 (-0.58, -0.38)**** |  |
|  | additional decline: incident AF at 50y | -0.18 (-0.39, 0.03) | -0.08 (-0.23, 0.06) | -0.10 (-0.26, 0.06) |  |
| **70 years** | **Decline between 55-70 year, NO AF** | ***-0.47 (-0.58, -0.36)**** | ***-0.42 (-0.49, -0.35)**** | ***-0.56 (-0.65, -0.48)**** |  |
|  | additional decline: incident AF at 55y | -0.12 (-0.30, 0.06) | -0.03 (-0.15, 0.09) | -0.05 (-0.18, 0.09) |  |
| **75 years** | **Decline between 60-75 years, NO AF** | ***-0.63 (-0.78, -0.47)**** | ***-0.49 (-0.58, -0.39)**** | ***-0.65 (-0.76, -0.53)**** |  |
|  | additional decline: incident AF at 60y | -0.04 (-0.21,-0.14) | -0.04 (-0.15, 0.07) | -0.06 (-0.19, 0.07) |  |
| **80 years** | **Decline between 65-80 years, NO AF** | ***-0.78 (-1.02, -0.55)**** | ***-0.56 (-0.69, -0.42)**** | ***-0.73 (-0.90, -0.56)**** |  |
|  | additional decline: incident AF at 65y | 0.07 (-0.22, 0.36) | -0.11 (-0.27, 0.06) | -0.14 (-0.35, 0.06) |  |
| **85 years** | **Decline between 70-85 years, NO AF** | ***-0.94 (-1.27, -0.61)**** | ***-0.62 (-0.81, -0.44)**** | ***-0.82 (-1.05, -0.58)**** |  |
|  | additional decline: incident AF at 70y | 0.20 (-0.38, 0.79) | -0.23 (-0.54, 0.08) | -0.30 (-0.70, 0.11) |  |
| ***Interaction between AF duration and age, p*** | | ***0.39*** | ***0.21*** | ***0.30*** |  |

*p<0.05

^†^ Participants aged 45-69 years in 1997-1999 were followed until 2012-2013, mean follow-up 14.7 years. Estimates are for decline over 15 years.

Analysis uses age as the time-scale; adjusted for sex, education, ethnicity, alcohol consumption, smoking, physical activity, diet, diabetes,

hypertension, heart failure, CVD (stroke or CHD), CVD medication at baseline (1997-1999), and time-dependent CVD (1997-2013).
